# Extracellular Adenosine (eAdo) - A_2B_ Receptor Axis Inhibits in Nlrp3 Inflammasome-dependent Manner Trafficking of Hematopoietic Stem/progenitor Cells

**DOI:** 10.1007/s12015-022-10417-w

**Published:** 2022-07-23

**Authors:** Arjun Thapa, Ahmed Abdelbaset-Ismail, Vira Chumak, Mateusz Adamiak, Katarzyna Brzezniakiewicz-Janus, Janina Ratajczak, Magdalena Kucia, Mariusz Z. Ratajczak

**Affiliations:** 1 Stem Cell Institute at James Graham Brown Cancer Center, University of Louisville, 500 S. Floyd Street, Rm. 107, 40202 Louisville, KY USA; 2 Department of Surgery, Anesthesiology and Radiology, Faculty of Veterinary Medicine, Zagazig University, Zagazig, Egypt; 3 Center for Preclinical Studies and Technology, Department of Regenerative Medicine, Medical University of Warsaw, Warsaw, Poland; 4 Department of Hematology, Multi-specialist Hospital Gorzow Wlkp, University of Zielona Gora, Zielona Gora, Poland

**Keywords:** Purinergic signaling, Adenosine, P1 receptors, Innate immunity, Nlrp3 inflammasome, Heme oxygenase-1, cAMP, NRF-2, Stem cell homing and engraftment

## Abstract

We postulated that mobilization, homing, and engraftment of hematopoietic stem/progenitor cells (HSCPs) is facilitated by a state of sterile inflammation induced in bone marrow (BM) after administration of pro-mobilizing drugs or in response to pre-transplant myeloablative conditioning. An important role in this phenomenon plays purinergic signaling that by the release of extracellular adenosine triphosphate (eATP) activates in HSPCs and in cells in the hematopoietic microenvironment an intracellular pattern recognition receptor (PPR) known as Nlrp3 inflammasome. We reported recently that its deficiency results in defective trafficking of HSPCs. Moreover, it is known that eATP after release into extracellular space is processed by cell surface expressed ectonucleotidases CD39 and CD73 to extracellular adenosine (eAdo) that in contrast to eATP shows an anti-inflammatory effect. Based on data that the state of sterile inflammation promotes trafficking of HSPCs, and since eAdo is endowed with anti-inflammatory properties we become interested in how eAdo will affect the mobilization, homing, and engraftment of HSPCs and which of eAdo receptors are involved in these processes. As expected, eAdo impaired HSPCs trafficking and this occurred in autocrine- and paracrine-dependent manner by direct stimulation of these cells or by affecting cells in the BM microenvironment. We report herein for the first time that this defect is mediated by activation of the A_2B_ receptor and a specific inhibitor of this receptor improves eAdo-aggravated trafficking of HSPCs. To explain this at the molecular level eAdo-A_2B_ receptor interaction upregulates in HSPCs in NF-kB-, NRF2- and cAMP-dependent manner heme oxygenase-1 (HO-1), that is Nlrp3 inflammasome inhibitor. This corroborated with our analysis of proteomics signature in murine HSPCs exposed to eAdo that revealed that A_2B_ inhibition promotes cell migration and proliferation. Based on this we postulate that blockage of A_2B_ receptor may accelerate the mobilization of HSPCs as well as their hematopoietic reconstitution and this approach could be potentially considered in the future to be tested in the clinic.

**Graphical Abstract:**

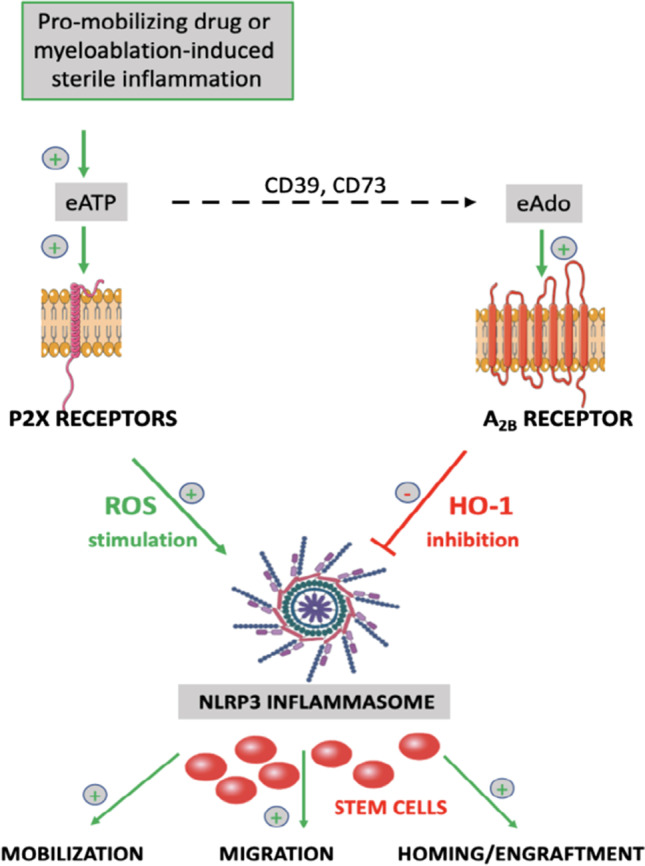

**Supplementary Information:**

The online version contains supplementary material available at 10.1007/s12015-022-10417-w.

## Introduction

Extracellular adenosine (eAdo) is a metabolite of extracellular adenosine triphosphate (eATP) and an important mediator of purinergic signaling that activates a family of G-protein coupled P1 purinergic receptors [1–4]. The conversion of eATP to eAdo is mediated by cell surface ectonucleotidases CD39 and CD73 expressed in the hematopoietic microenvironment and on hematopoietic stem/progenitor cells (HSPCs) [1, 4–8]. The four P1 receptor subtypes A_1_, A_2A_, A_2B,_ and A_3_ are classified based on their ability to either stimulate or inhibit adenylate cyclase activity [1, 2, 4, 9]. While A_1_ receptors coupled to G_i/o_ proteins decrease cyclic AMP (cAMP) levels, the A_2_ adenosine receptors coupled to G_s_ proteins stimulate adenylate cyclase activity [2, 3, 5, 9]. It has been demonstrated in vascular smooth cells that cAMP similarly as NRF2 increases the expression of anti-inflammatory enzyme heme oxygenase-1 (HO-1) that as reported inhibits cell migration [10, 11]. However, in the case of HSPCs, a type of P1 receptor involved in inhibition has not been identified so far.

The concentration of eAdo increases up to 4 times during inflammation and hypoxia in affected tissues and eAdo displays several cardio-vascular and central nervous system-mediated effects affecting heart rate, endothelial and smooth muscle cell relaxation, and decreasing dopamineragic activity in the brain and excitation of central neurons [12–14]. Interestingly, eAdo has been also shown to be involved in the emergence of hematopoiesis in zebra fish and plays an evolutionary conserved role in the first steps of hematopoietic stem progenitor cells (HSPCs) formation in vertebrates [15]. These effects were mediated after stimulation of A_2B_ receptors. Moreover, in adult mice, A_1_ and A_3_ receptors play a homeostatic opposite role in suppressing or stimulating hematopoiesis, respectively [2, 16, 17]. As reported selective activation of A_3_ receptors promotes hematopoiesis by acting on differentiated hematopoietic progenitors [2, 18, 19].

For many years we are interested in mechanisms that regulate the mobilization of HSPCs from bone marrow (BM) into peripheral blood (PB) as well as homing from PB to BM and engraftment after transplantation [20–26]. Recently we focused on the role of purinergic signaling and demonstrated that eATP-dependent activation of purinergic signaling promotes the egress of HSPCs from BM into PB as well as directs their homing and engraftment [6, 7, 23]. These biological effects of eATP depend on the stimulation of purinergic P2X receptors and are mediated by activation of intracellular pattern recognition receptor (PRR) known as Nlrp3 inflammasome [22, 24, 27–30].

In our previous work, we have shown that eAdo inhibits the egress of HSPCs from BM into PB in response to pharmacological mobilization after administration of granulocyte colony stimulating factor (G-CSF) and CXCR4 receptor antagonist AMD3100 [6]. We have also postulated that this negative effect on migration of HSPCs is mediated in heme oxygenase-1 (HO-1)-dependent manner inhibition of Nlrp3 inflammasome [31, 32]. To support this notion, a decrease in eAdo level in BM microenvironment after blocking CD39 and CD73 ectonucleotidases improved pharmacological mobilization of murine HSPCs [7]. However, we did not address in previous studies which of eAdo specific P1 receptors were involved in this defect. Moreover, to shed more light on the effects of eAdo on HSPCs trafficking we become also interested in its role in a process opposite to mobilization that is homing and engraftment of transplanted cells. This was justified by our data that both mobilizations similarly like homing/engraftment are regulated in BM by purinergic signaling and Nlrp3 inflammasome.

Herein, we provide further support on the role of purinergic signaling mediated sterile inflammation in regulating mobility of HSPCs, and provide an evidence that eAdo as an anti-inflammatory mediator plays an inhibitory role. We also identified that the eAdo effect is mediated via A_2B_ receptor that in NF-kB-, NRF2- and cAMP-dependent manner induces an intracellular level of HO-1 to inhibit Nlrp3 inflammasome. Furthermore, analysis of proteomics signature in murine HSPCs exposed to eAdo supported that A_2B_ inhibition improves cell migration and proliferation. Based on this, inhibition of A_2B_ by a small molecular antagonist may positively affect the mobilization of HSPCs and their reconstitution after transplantation.

## Materials and Methods

### Animals

Pathogen-free 6–8 weeks old C57BL/6 J mice (WT) and B6.129S1-Nt5e^tm1Lft^/J (CD73-KO) were purchased at least 2 weeks before experiments from Jackson Laboratory, Maine, USA. Animal studies were approved by the University of Louisville (Louisville, KY, USA) and the Medical University of Warsaw (Warsaw, Poland).

#### Human Umbilical Cord Blood

Clinical-grade umbilical cord blood (UCB) research units were obtained from the Cleveland Cord Blood Center (Cleveland, OH). This study was performed in accordance with the guidelines and approval of the Institutional Review Board at the University of Louisville School of Medicine (Louisville, Kentucky).

### Drug Administration in Animals

Mice were injected with G-CSF (45 µg/kg daily; Amgen, CA, USA) for 4 days by subcutaneous injection (SC) or AMD3100 (1 mg/kg once; Sigma-Aldrich, MO, USA) for 1 day or adenosine receptor P1 antagonists, A_2A_ inhibitor (SCH442416; 3 mg/kg daily, Tocris, Minneapolis, USA) for 7 days or A_2B_ inhibitor (PSB1115; 3 mg/kg daily, Tocris, Minneapolis, USA) for 7 days via intraperitoneal injection (IP). The stock solution of both these inhibitors was prepared in DMSO. Corn oil was used to further solubilize the A_2A_ inhibitor. In mobilization studies the G-CSF groups of mice were injected with A_2B_ inhibitor (PSB1115) or A_2A_ (SCH442416) or G-CSF or a mixture of G-CSF and A_2A_ inhibitor or a mixture of G-CSF and A_2B_ inhibitor. A_2A_ inhibitor was administered separately in a 50 µl injection volume due to its poor solubility in an aqueous solution and high viscosity. The G-CSF groups of mice received the first dose of G-CSF injection on day 4th after completing 3 doses (daily once) of A_2A_ or A_2B_ inhibitor. Mice in these groups were sacrificed 6 h after the final injection. The inhibitors were administered similarly in AMD3100 groups. The AMD3100 groups of mice were sacrificed one hour after the final dose of injection [6, 22]. In homing/engraftment experiments, A_2A_ or A_2B_ inhibitor or adenosine (5 mg/kg, IP, every 2nd day, dependent on the experiment from 3 to 9 doses; Tocris, Bristol, UK) were injected to transplant recipient mice groups.

### Isolation of Peripheral Blood and Bone Marrow

Peripheral blood (PB) and bone marrow (BM) samples were collected from all groups of mice [6, 22]. Briefly, a 50 µl of PB was withdrawn from retro-orbital plexus or tail-vein injection in EDTA-coated microvette tubes (Sarstedt Inc., Newton, NC, USA) for hematologic analysis. The blood samples were run on HemaVet 950FS hematology analyzer (Drew Scientific Inc., Oxford, CT, USA) within 2 h of collection. PB was also collected from the posterior vena cava (with a 25-gauge needle and 1-ml syringe containing 250 U heparin). The red blood cells (RBCs) were lysed by hypotonic lysis buffer (BD Biosciences, CA, USA) and PB mononuclear cells (PBMNCs) were obtained by centrifugation. BM mononuclear cells (BMMNCs) were also flushed from tibias and femurs. RBCs present in BM were lysed, washed, and BMMNCs were resuspended in PBS or 2% FBS containing RPMI media as necessary.

### Migration Assay

The RPMI-1640 medium containing 0.5% BSA was used for the Transwell migration assay performed as described [6, 22]. A 650-µl volume of medium with or without stromal-derived factor 1 (SDF-1, 5 ng/ml), sphingosine-1-phosphate (S1P, 0.1 µM), ceramide-1-phosphate (C1P, 100 µM), adenosine triphosphate (ATP, 0.25 µg/ml). A_2B_ inhibitor (PSB1115) or A_2A_ (SCH442416) or to ARL67156 (10 mM) CD73 inhibitor or AMPCP (10 mM) CD39 inhibitor were added to the lower chamber of a Costar Transwell 24-well plate (Corning Inc., Corning, NY, USA). An aliquot (1 × 10^6^ cells per 100 µl) of murine or human BMMNCs was loaded onto the upper chamber separated by a 5-µm pore size insert. The plate was incubated for 3 h at 37 °C in a 5% CO_2_ incubator. Following incubation, an aliquot of cells from the lower chamber was harvested and scored by FACS analysis. Briefly, the cells were gated according to their forward-scatter (FSC) and side-scatter (SSC) parameters and counted during a 30-s acquisition at a high flow rate. The rest of the BMMNCs recovered from the lower chamber were resuspended in a human methylcellulose base medium provided by the manufacturer (R&D Systems), supplemented with murine GM-CSF (25 ng/ml) and IL-3 (10 ng/ml) for determining the number of CFU-GM colonies. Next, the cultures were incubated for 7 days at 37 °C in a 5% CO_2_ incubator. The colony-forming unit-granulocyte/monocyte (CFU-GM) colonies were then counted under an inverted microscope.

### Mobilization Analysis

PB blood withdrawn from the retro-orbital plexus was used for white blood cell (WBC) counts. The blood samples were analyzed by HemaVet 950FS hematology analyzer as described [21, 22]. PB blood isolated from vena cava was lysed and PBMNCs were isolated as motioned above. PBMNCs were resuspended RPMI media containing 2% FBS. The PBMNCs were cultured on a human methylcellulose base medium (R&D Systems, Minneapolis, MN, USA) supplemented with 25 ng/ml recombinant murine granulocyte/macrophage colony-stimulating factor (mGM-CSF) and 10 ng/ml recombinant murine interleukin 3 (mIL-3). Cells were incubated for 7 days (37 °C, 95% humidity, and 5% CO_2_). The colony-forming unit-granulocyte/monocyte (CFU-GM) was scored using a simple inverted microscope (Olympus, Center Valley, PA, USA). In addition, PBMNCs were also stained for Lin^−^/Sca-1^+^/c-Kit^+^ (SKL) cells analysis using the following monoclonal antibodies: FITC–anti-CD117 (also known as c-Kit, clone 2B8; BioLegend, CA, USA), PE–Cy5–anti-mouse Ly-6 A/E (also known as Sca-1, clone D7; eBioscience, San Diego, USA), and anti-mouse lineage-marker antibodies, including anti-CD45R (also known as B220, clone RA3-6B2), anti-Ter-119 (clone TER-119), anti-CD11b (clone M1/70), anti-T cell receptor β (clone H57-597), anti-Gr-1 (clone RB6-8C5), and anti-TCRγδ (clone GL3) conjugated with PE (BD Biosciences, San Jose, CA, USA). Staining was performed in RPMI-1640 medium containing 2% FBS. All monoclonal antibodies were added at saturating concentrations, and the cells were incubated for 30 min on ice, washed twice, and analyzed using an LSRII flow cytometer (BD Biosciences, CA, USA).

### BMMNCs Transplantation

The short-term transplant homing, early engraftment, and hematopoietic recovery analysis were performed in wild-type (WT) or CD73-KO mice irradiated with a lethal dose of γ-irradiation (1000 cGy) as described [22, 23]. WT or CD73-KO transplant recipient mice were irradiated 24 h before the transplantation procedure. Two different sets of transplantation experiments were performed in irradiated mice. In the first set of the experiments, WT mice but not CD73-KO were injected with vehicles or adenosine or A_2A_ or A_2B_ inhibitor (3 mg/kg daily for 7 days as described above) prior to the irradiation procedure. These groups of mice were transplanted with HSPCs isolated from WT or CD73-KO mice by tail vein injection. In the second set of experiments, recipient mice were not injected with A_2A_ or A_2B_ inhibitors prior irradiation. The recipient WT mice received WT mice HSPCs treated with vehicle or A_2A_ or A_2B_ inhibitor (10 µM each) for 30 min or HSPCs (untreated with inhibitor) from CD73-KO mice by tail vein injection. The CD73-KO recipient mice were transplanted with HSPCs (untreated) from WT mice.

For the short-term homing experiment, BMMNCs (5 × 10^6^/100 µl) from WT mice were labeled PKH-67 with a green fluorescent dye (Sigma-Aldrich, St Louis, MO, USA) and transplanted into the recipient in mice. The next day, femurs were isolated by Ficoll-Paque density-gradient centrifugation. The cells were divided into two aliquots, one of which was analyzed by flow cytometer for PKH-67 positive cells and the other aliquot was resuspended in a human methylcellulose base medium supplemented with mGM-CSF (25 ng/ml) and IL-3 (10 ng/ml). The cultures were incubated for 7 days at 37 °C in a 5% CO_2_ incubator. The CFU-GM colonies were then counted using a simple inverted microscope. Similarly, early engraftment and hematopoietic recovery analysis were performed in irradiated recipient mice using unlabeled BMMNCs transplants. Mice in the engraftment assay groups were transplanted with 1.5 × 10^5^ (100 µl) BMMNCs. Twelve days after the transplantation, femurs were flushed and cultured for CFU-GM colonies as described above. Spleen from these groups of mice was also removed and fixed in Telesyniczky’s solution. CFU-S colonies on the surface of the spleen were counted using a magnifying glass.

To perform a hematopoietic recovery study, recipient mice were transplanted with 7.5 × 10^5^ (100 µl) BMMNCs. At the time intervals indicated, a 50 µl of PB was drawn into EDTA-coated Microvette tubes (Sarstedt Inc., Newton, NC, USA) to count white blood cells (WBC) and platelet (PLT).

### Adhesion Assay

The adhesion assay was formed in a 96-well plate coated with fibronectin (10 µg/ml) at 4 °C overnight and treated with a 0.05% BSA blocking medium for 2 h. BMMNCs from WT mice were suspended in an RPMI-1640 medium containing 0.5% BSA with a density of 5 × 10^4^/100 µl as described [26]. The cells maintained in a quiescence state for 3 h at 37 °C were incubated in the presence or absence of A_2B_ inhibitor (PSB1115) or A_2A_ (SCH442416) or to ARL67156 (10 mM) CD73 inhibitor or AMPCP (10 mM) CD39 inhibitor for 1 h at 37 °C. Cells were placed in the fibronectin pre-coated plate. After a 5 min incubation at room temperature, non-adherent cells were washed, and the adherent cells were counted using an inverted microscope.

### qRT-PCR

BMMNC from WT mice or human UCB HSPCs were isolated from human UCB and murine BM [22, 24]. Briefly, human UCB was separated on Ficall-Paque (GE Healthcare, Chicago, IL, USA), and derived MNCs were stained with the following antibodies: FITC lineage marker-specific: anti-CD235a, anti-CD2, anti-CD3, anti-CD14, anti-CD16, anti-CD19, anti-CD24, anti-CD56, anti-CD66b; PE-Cy7 anti-CD45 and APC anti-CD34 (all BD Biosciences, San Jose, CA, USA). Lin^−^CD45^+^CD34^+^ (HSPCs) population of cells was sorted on MoFlo Astrios Cell Sorter (Beckman Coulter, Brea, CA, USA). Murine femurs and tibias were flushed and cells were lysed. Obtained MNCs were stained with the following antibodies: APC–anti-mouse Ly-6 A/E (also known as Sca-1, BioLegend, San Diego, CA, USA), PE-Cy7–anti-CD45 and anti-mouse lineage-marker antibodies, including anti-CD45R, anti-Ter-119, anti-CD11b, anti-T cell receptor β, anti-Gr-1, and anti-TCRγδ conjugated with PE (BD Biosciences, San Jose, CA, USA). Lin^−^Sca1^+^CD45^+^ cells were sorted. Total RNA from human and murine MNCs and sorted HSPCs was isolated using RNeasy Mini kit (Qiagen Inc., Germany), and reverse transcribed with iScript reverse transcriptase (Bio-Rad, Hercules, CA, USA). Evaluation of the target genes was then performed using iTaq Universal SYBR Green Supermix (Bio-Rad, Hercules, CA, USA) and specific primers. The samples were run on an Bio-Rad CFX96 qPCR Instruments detection system (Bio-Rad, Hercules, CA, USA). The PCR cycling conditions were 95 °C (30 s), 40 cycles at 95 °C (5 s), and 60 °C (30 s) plus the melting curve. According to melting point analysis, only one PCR product was amplified under these conditions. The relative quantity of a target gene, normalized to the β2-microglobulin gene as the endogenous control and relative to a calibrator (Adora A_1_, MNCs) was expressed as 2^−ΔΔCt^. PCR products were visualized on 2% agarose gels. The following primer pairs were used for analysis: Human: Adora A_1_ – F:TGCGAGTTCGAGAAGGTCATC, R:GAGCTGCTTGCGGATTAGGTA; Adora 2_ A_ – F:CGA GGGCTAAGGGCATCATTG, R:CTCCTTTGGCTGACCGCAGTT; Adora 2_B_ – F:TAAAAGT TTGGTCACGGGGACCCGA, R:TTCACAAGGCAGCAGCTTTCATTCGT; Adora 3 - F:TAC ATCATTCGGAACAAACTC, R:GTCTTGAACTCCCGTCCATAA; β-2microglobuline - F:TG ACTTTGTCACAGCCCAAGATA, R:AATGCGGCATCTTCAAACCT. Murine: Adora A_1_ – F:TTGTGGTAGGCCTGACACCCATGT, R:GCCGTTGGCTATCCA GGCTTGTT; Adora 2_ A_ – F: TCCTCGGTGTACATCATGGTGGAGC, R:CGAAGAAGTTGGT GACGTTCTGCAGG; Adora 2_B_ – F: TGCATTACAGACCCCCACCAACTACTTT, R:AAGAG GCTAAAGATGGAG CTCTGTGTGAG; Adora 3 - F:TCAGGTGTTGAGCTGGAGACAGCTT, R:ATGAGTTGGTT TCCTGTATCCTTCAGGGC; mHO-1–F: CCTCACAGATGGCGTCACTT, R: GCTGATCTGGGGTTTCCCTC; β-2microglobuline - F:TGCTGCTTGTCTC ACTGAC, R:GGAT TTCAATGTGAGGCGG.

### Western Blot Analysis

The quiescent cells were treated in vitro for 1 h at 37oC with the vehicle, adenosine alone, adenosine plus SCH442416, or adenosine plus PSB1115 in a serum-free RPMI 1640 medium. Cells were then harvested, centrifuged, washed, and lysed by RIPA lysis buffer supplemented with protease and phosphatase inhibitors (Santa Cruz Biotechnology, TX, USA). The protein concentration was measured by using a BCA protein assay kit (Pierce, Rockford, IL, USA). Proteins (40 µg of each sample) were then separated on a 4–12% SDS-PAGE gel and transferred to a polyvinylidene fluoride (PVDF) membrane (Bio-Rad). The membranes were blocked with 2.5% BSA in Tris-buffered saline containing 0.1% Tween (TBST buffer) for 1 h at room temperature. After washing with TBST, the membranes were incubated with rabbit anti-HO-1 polyclonal antibody and Rabbit anti-NRF2 (E5F1A) monoclonal antibody (diluted 1:1000; Cell signaling Technology, USA) overnight at 4 °C. Blots were also stripped and then probed with rabbit anti-β-actin monoclonal antibody (diluted 1:1000; Novus Biologicals, CO, USA). Phosphorylation of the intracellular the nuclear factor (NF)-kappa B p65(RelA) was detected, after 5 min stimulation, by incubating the membranes overnight at 4 °C with phosphospecific rabbit anti-p-NF-kB p65 monoclonal antibody (Ser536; clone no. 93H1, diluted 1:1000; Cell Signaling). Afterward, the membranes were incubated with horseradish peroxidase (HRP)-conjugated goat anti-rabbit IgG secondary antibody (Santa Cruz Biotech, Santa Cruz, CA, USA, 1:5000). To confirm equal protein loading in all lanes, the blots were stripped using stripping buffer (Thermo Scientific) and then reprobed with appropriate rabbit anti-NF-kB p65 monoclonal antibody (clone no. D14E12; Cell Signaling). Membranes were treated with an enhanced chemiluminescence (ECL) reagent (Amersham Life Sciences, PA, USA) and subsequently exposed to film (Hyperfilm; Amersham Life Sciences) as described [6, 31].

### Quantification of the Intracellular cAMP

Quiescent cells were treated with the targeted compounds in serum-free medium and then washed with cold PBS. After the cell were lysed, and centrifuged at 4oC, the supernatants were examined for the intracellular cAMP levels according to the Cyclic AMP XP® Assay Kit protocol (Cell Signaling Technology, Inc, MA).

### Glow Assay to Measure Activation of Nlrp3 Inflammasome

To measure the activity of caspase-1 in cells Caspase-Glo® 1 Inflammasome Assay (Promega, USA) was employed, and analyses were performed according to the manufacturer’s protocols. Samples of control and cells exposed to ATP, ATP + Ado and ATP + Ado + A_2A_ or A_2B_ inhibitors were collected 10 × 10^5^ of BM MNC were plated in 96 wells plate. Caspase-Glo® 1 Reagent or Caspase-Glo® 1 YVAD-CHO Reagent were added (100 µl/well), and luminescence was measured using a GloMax 9301 Multi Detection System after 90 min [22].

### Proteomics Analysis

Murine SKL (Sca-1^+^/c-Kit^+^/Lin^−^) cells were sorted using cell sorter MoFlo Astrios (Beckmam Coulter) from freshly isolated bone marrow [21]. 1 × 10^4^ SKL cells were treated in serum-free RPMI for 12 h under the following conditions: (1) 100 µM adenosine (Ado), (2) 100 µM adenosine in co-treatment with 20 µM Sch442416 and (3) 100 µM adenosine in co-treatment with 10 µM PSB1115. Then cells were collected and lyzed in RIPA buffer. Proteins were precipitated with ice-cold acetonitrile LC-MS grade (Merck Millipore, USA), centrifuged at 18,000 x g for 30 min at -10 °C, and dried for 10 min at RT using Eppendorf Concentrator plus (Eppendorf, Germany). Then proteins were re-dissolved in 40 mM ammonium bicarbonate solution, reduced with 20 mM DTT solution, and alkylated with 40 mM iodoacetamide solution (Sigma Aldrich, USA). In-solution trypsin digestion was performed overnight at 37 °C with shacking and the digestion was stopped by adding 0.1% formic acid. LC-MS analysis was carried out with the use of nanoUHPLC (nanoElute, Bruker) coupled by CaptiveSpray (Bruker) to ESI-Q-TOF mass spectrometer (Compact, Bruker). Two-Column separation method was used, i.e. pre-column (300 μm x 5 mm, C18 PepMap 100, 5 μm, 100Å, Thermo Scientific) and Bruker FIFTEEN separation column with CSI fitting (75 μm x 150 mm, C18 1.9 μm) in gradient 2% B to 35% B in 30 min with the 300 nL/min flow rate. Following mobile phases were used: A – 0.1% formic acid in water; B – 0.1% formic acid in ACN. Ionization of the samples were carried out at a gas flow of 3.0 L/min, temperature of 150 °C and voltage of the capillary 1600 V. The quadrupole energy was set to 5.0 eV and collision chamber energy 7.0 eV with an ion transfer time of 90 µs. The ions were analyzed in the positive polarity mode in the range 150–2200 m/z, with the acquisition frequency of the 4 Hz spectrum, as well as with the autoMS/MS system. The collected spectra were analyzed and calibrated (Na Formate) in DataAnalysis software (Bruker) and then, after extracting the peak list, identified in ProteinScape (Bruker) using the MASCOT server. Proteins were identified using the online SwissProt and NCBI_prot databases, and their annotation and biological significance were identified using Reactome.org.

### Statistical Analysis

All results are presented as mean ± SEM. Statistical analysis of the data was done using Student’s *t*-test for unpaired samples, with p ≤ 0.05 considered significant.

## Results

### HSPCs Express A_2A_ and A_2B_ Receptors for eAdo and Migration of These Cells is Inhibited in eAdo-A_2B_ Receptor-dependent Manner

**Fig. 1:**
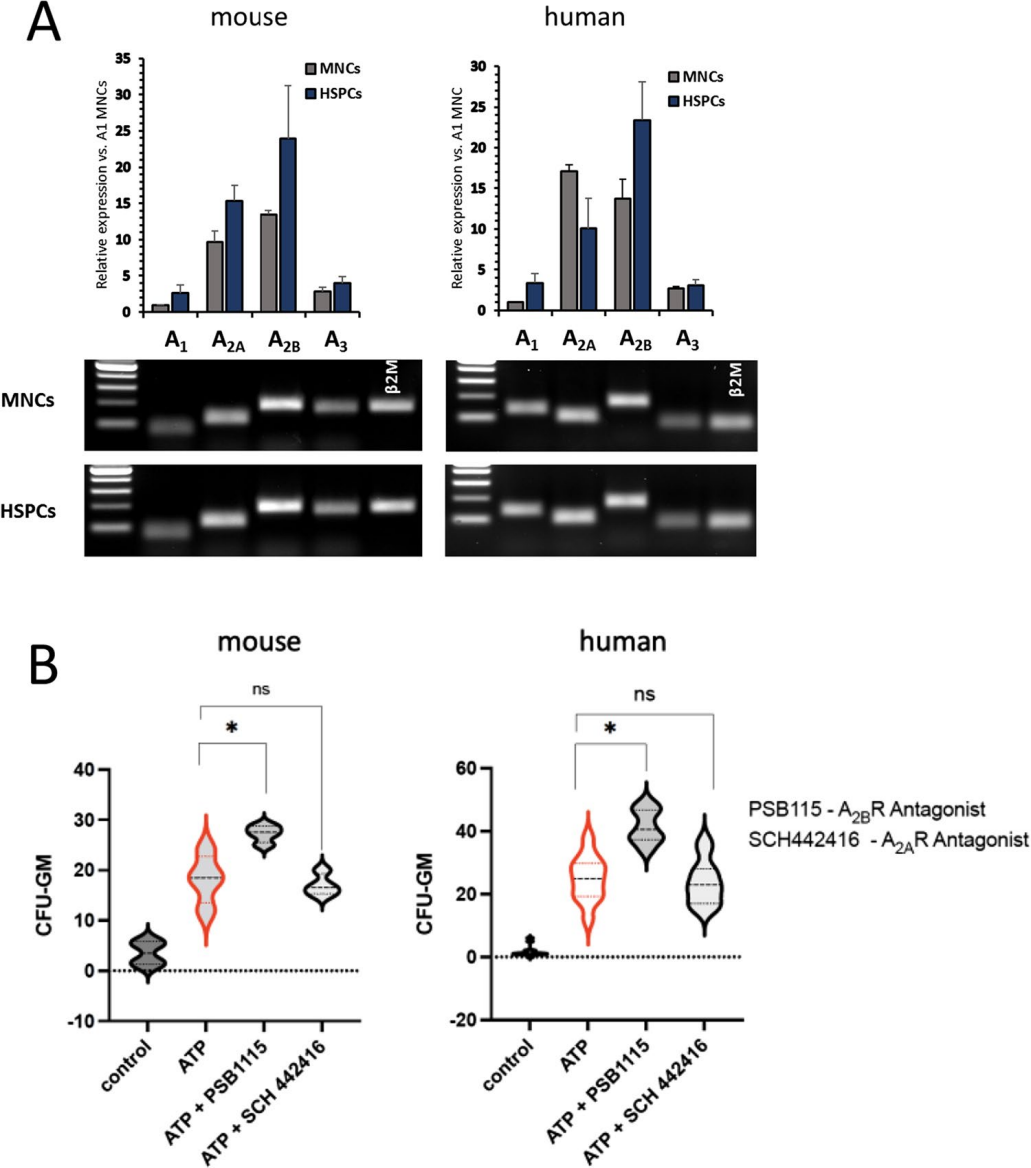
Expression of P1 receptors on murine and human HSPCs and their chemotactic responsiveness. Expression levels of Adora A_1_, A_2A_, A_2B_ and A_3_ receptors mRNA (**Panel A**) were evaluated by qRT-PCR analysis in human UCB (left) and murine BM derived (right) MNCs and HSPCs. The expression was normalized to β-2microglobuline Ct value and shown relative to A_1_ receptor mRNA. The data are presented as means ± SD from 3 separate analysis. Samples were separated on 2% agarose gels stained with ethidium bromide to visualize qRT-PCR reaction products. Representative image is shown. (**Panel B**) Transwell migration assay (pore size 5 μm) with WT murine BMMNC (left panel) and human UCBMNC (right panel). Data shows the number of CFU-GM colonies migrating to ATP (0.25 µg/ml) alone or in the presence of A_2A_ inhibitor (SCH442416, 10 µM) or A_2B_ inhibitor (PSB1115, 10 µM). Number of CFU-GM colonies was assayed in methylcellulose cultures after plating cells that migrated to lower Transwell chambers. The data are presented as means ± SE. Unpaired Student’s *t*-test was used for the determination of significance (*p ≤ 0.05 and #p ≤ 0.005)

In our previous work we noticed that eAdo inhibits migration of murine and human HSPCs [6]. This prompt us to address which one of P1 receptors is involved in this phenomenon and we first phenotyped murine and human HSPCs for expression of P1 receptors and noticed that these cells highly express mRNA for A_2A_ and A_2B_ members of P1 purinergic receptor family (Fig. 1A). To address a role of these receptors in inhibiting migration of HSPCs we employed specific small molecular antagonists of A_2A_ and A_2B_ receptors SCH442416 and PSB115, respectively. Figure 1B shows that inhibition of A_2B_ receptor but not A_2A_ improved significantly eATP gradient-dependent TransWell migration of murine (left panel) and human (right panel) clonogeneic CFU-GM cells. This was also confirmed after replacing eATP by more specific A_2B_ agonist BAY60:6083 (not shown). This data supports a major involvement of A_2B_ receptor in eAdo mediated inhibition of HSPCs migration.

### eAdo Inhibits Mobilization of HSPCs in A_2B_ Receptor-dependent Manner

**Fig. 2:**
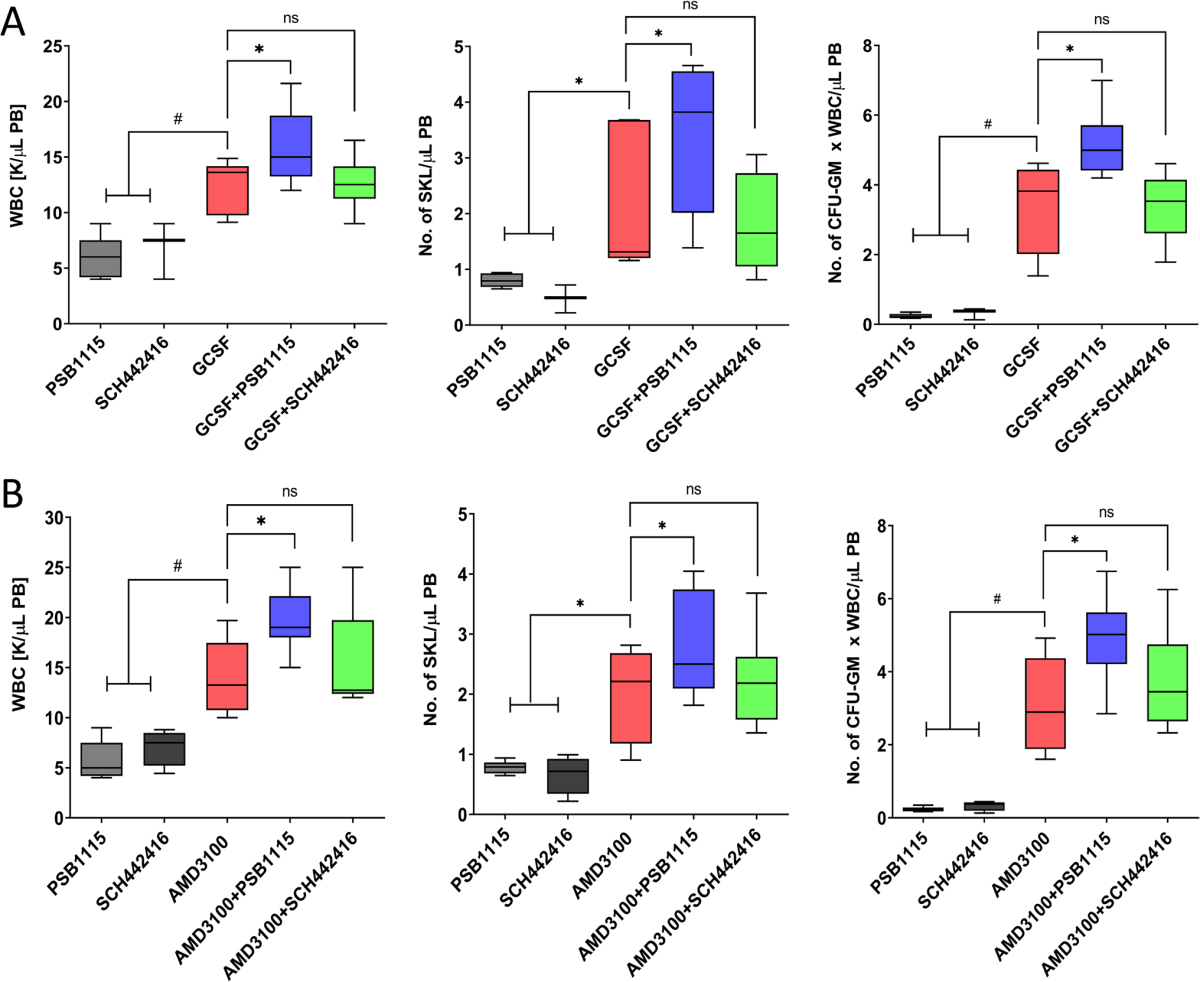
eAdo inhibits pharmacological mobilization of HSPCs in A_2B_-dependent manner. (**Panel A**) Data show an enhanced mobilization of WT mice injected with G-CSF (45 µg/kg for 4 days) in the presence A_2B_ but not A_2A_ inhibitor as compared to G-CSF alone. Mice were injected with A_2B_ inhibitor PSB1115 (3 mg/kg for 7 days daily) alone or A_2A_ inhibitor SCH442416 (3 mg/kg for 7 days daily) alone or G-CSF (45 µg/kg for 4 days) alone or G-CSF in the presence of A_2B_ or A_2A_ inhibitor. Mice were sacrificed 6 h after the final dose of the injections and we evaluated number of WBC circulating in PB (left panel), number of SKL cells (middle panel) and number of clonogenic progenitors (right panel). (**Panel B**) Data show an enhanced mobilization of WT mice injected with AMD3100 (1 mg/kg injected once) in the presence of A_2B_ but not A_2A_ inhibitor as compared to G-CSF alone. Mice were injected with A_2B_ inhibitor PSB1115 (3 mg/kg for 7 days daily) alone or A_2A_ inhibitor SCH442416 (3 mg/kg for 7 days daily) alone or AMD3100 (1 mg/kg once) alone or in the presence of A_2B_ or A_2B_ inhibitor. Mice were sacrificed 1 h after injections and we evaluated number of WBC circulating in PB (left panel), number of SKL cells (middle panel) and number of clonogenic progenitors (right panel). The data are presented as means ± SE, and an unpaired Student’s *t*-test was used for the determination of significance (*p ≤ 0.05; #p ≤ 0.005)

We observed in our previous study a negative effect of eAdo on pharmacological mobilization of murine HSPCs and become interested which of the P1 receptors is involved in this phenomenon. To address this question based on data shown in Fig. 1 we exposed mice before G-CSF or AMD3100 mobilization to small molecular antagonists of A_2A_ and A_2B_ receptors SCH442416 and PSB1115, respectively. Figure 2 shows that inhibition of A_2B_ but not A_2A_ receptor improves pharmacological mobilization of HSPCs from BM into PB. Based on this pharmacological blockade of A_2B_ receptor by small molecular antagonist could become a novel strategy to enhance egress of HSPCs from BM into PB.

### eAdo Inhibits in A_2B_-dependent Manner Homing and Engraftment of Murine HSPCs

**Fig. 3:**
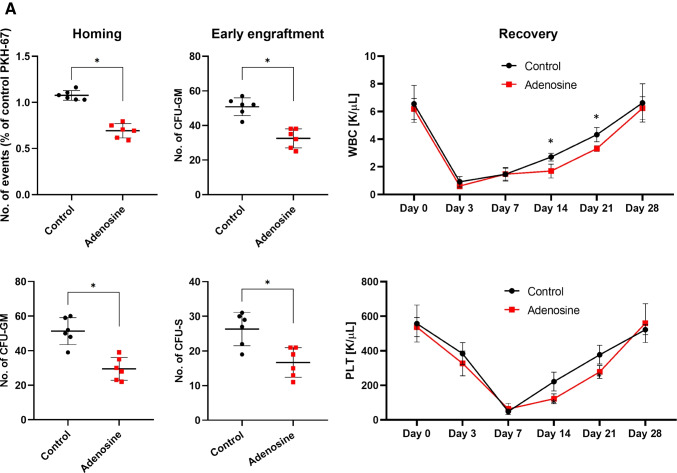

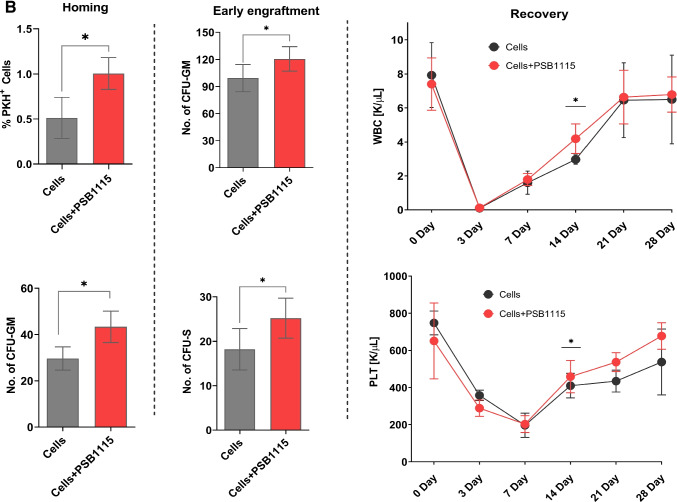

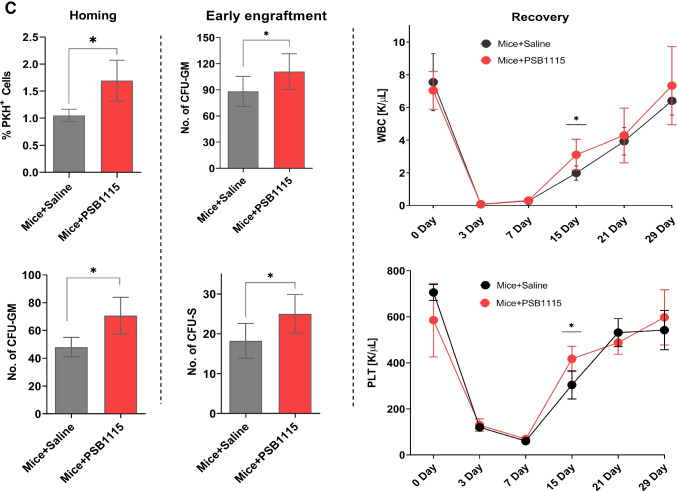
eAdo inhibits homing and engraftment of transplanted BMMNC in A_2B_ receptor-dependent manner. **Panel A.** Wild type (WT) BMMNCs were exposed or not (controls) before transplantation in vitro to eAdo and transplanted along with normal control WT BMMNCs) into normal animals. Left panel shows decreased 24 h homing of transplanted BMMNC as assayed by enumeration of PKH-67 labeled cells and number of CFU-GM progenitors in BM of transplanted mice. Middle panel – number of CFU-GM progenitors in BM and CFU-S in spleens of transplanted mice at day 11 after injection of BMMNC. Right panel – recovery of WBC and platelets in PB of transplanted animals. The data are presented as means ± SE, and an unpaired Student’s *t*-test was used for the determination of significance (*p ≤ 0.05). **Panel B**. Wild type (WT) mice were transplanted with BMMNC that before transplantation were exposed or not to A_2B_ inhibitor. Left panel shows decreased 24 h homing of transplanted BMMNC as assayed by enumeration of PKH-67 labeled cells and number of CFU-GM progenitors in BM of transplanted mice. Middle panel – number of CFU-GM progenitors in BM and CFU-S in spleens of transplanted mice at day 11 after injection of BMMNC. Right panel – recovery of WBC and platelets in PB of transplanted animals. The data are presented as means ± SE, and an unpaired Student’s *t*-test was used for the determination of significance (*p ≤ 0.05). **Panel C**. Wild type (WT) mice were exposed or not (controls) before transplantation to A_2B_ inhibitor and transplanted with normal BMMNCs. Left panel shows decreased 24 h homing of transplanted BMMNC as assayed by enumeration of PKH-67 labeled cells and number of CFU-GM progenitors in BM of transplanted mice. Middle panel – number of CFU-GM progenitors in BM and CFU-S in spleens of transplanted mice at day 11 after injection of BMMNC. Right panel – recovery of WBC and platelets in PB of transplanted animals. The data are presented as means ± SE, and an unpaired Student’s *t*-test was used for the determination of significance (*p ≤ 0.05)

After we learned that eAdo inhibits migration of HSPCs and their egress from BM (Figs. 1 and 2) we become interested in its effect on reverse phenomenon that is homing and engraftment of transplanted BM cells. First, BMMNCs from wild type (WT) mice were exposed or not (controls) in vitro before transplantation to eAdo and subsequently transplanted into lethally irradiated recipients. Figure 3A, demonstrates negative effect of eAdo on homing, early engraftment and hematopoietic reconstitution of transplanted animals. Homing was evaluated by the number of fluorochrome PKH67 labeled cells detected in BM of transplanted mice and the number of CFU-GM clonogeneic progenitors derived from donor BM cells. As demonstrated in Fig. 3A – left panel, mice exposed to eAdo show a ~ 30% reduction of BM homing of transplanted HSPCs as measured by the presence in BM of PKH67^+^ labeled cells and clonogeneic CFU-GM progenitors 24 h after transplantation. Similarly, we observed defective early 12 days engraftment of HSPCs in BM of mice exposed to eAdo as assayed by evaluating the presence of clonogeneic CFU-GM in BM of transplanted mice after 12 days as well as the number of 12 days spleen colonies (ang. Colony-forming units in the spleen; CFU-S) after transplantation (Fig. 3A – middle panel). Finally, normal mice were transplanted with cells exposed before transplantation to eAdo what resulted in delayed recovery of peripheral blood white cells and platelets counts (Fig. 3A – right panel).

Finally, we transplanted lethally irradiated mice exposed to eAdo and control non exposed to eAdo with syngeneic BMMNCs cells and followed the recovery of peripheral blood counts. Figure 3A – righty panel shows that eAdo pretreated transplant recipient mice have delayed recovery of peripheral blood leucocytes and platelets counts. This data indicates that eAdo is a negative regulator of homing and engraftment of HSPCs.

We were aware that eAdo could negatively affect homing and engraftment of HSPCs by involving two mechanisms. First, eAdo could be generated in autocrine dependent manner by migrating HSPCs as result of degradation in CD39 and CD73-dependent manner of eATP secreted from these cells [7, 8]. On the other hand eAdo could be also upregulated in BM conditioned for transplantation after processing eATP to eAdo as result of sterile inflammation [8, 21, 33]. The question in both cases remained which of the A_2_ receptors is crucial in negative effect of eAdo in homing and engraftment of transplanted HSPCs.

Therefore, as next step we exposed or not exposed BMMNC before transplantation to A_2B_ or A_2A_ inhibitors and evaluated homing, engraftment and hematopoietic recovery of transplanted mice. Figure 3B demonstrates that BMMNCs exposed before transplantation to A_2B_ inhibitor as well as recipient mice exposed before transplantation to A_2B_ inhibitor (Fig. 3C) displayed improved homing and engraftment of transplanted BMMNCs. At the same time homing of transplanted BMMNCs was not affected after inhibition of A_2A_ (Supplementary Fig. 1). The hematopoietic recovery of transplanted mice was also inhibited after administration of more specific agonist of A_2B_ receptor that is BAY60:6083 (Supplementary Fig. 2). This data supports a negative role of eAdo and its A_2B_ receptor in posttransplant recovery of mice and this inhibitory axis seems to operate both at level of transplanted cells as well as depends on changes in hematopoietic microenvironment in recipient mice.

### An Evidence That eAdo inhibits Nlrp3 Inflammasome in A_2B_-NF-kBp65-NRF2-cAMP-HO-1-dependent Manner

**Fig. 4:**
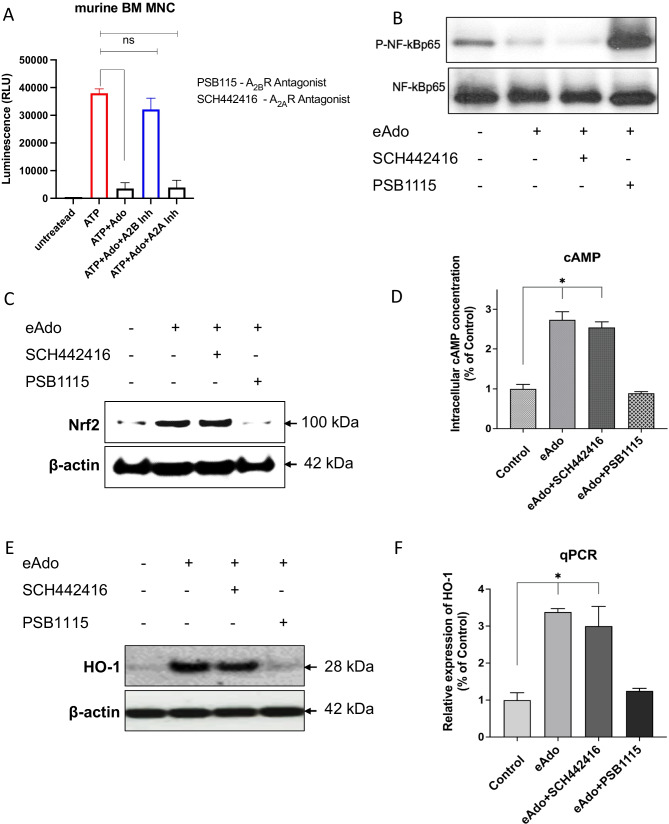
eAdo inhibits Nlrp3 inflammasome and this occurs in A_2B_-cAMP-NRF2_−_-HO-1 dependent manner. **Panel (A)** Effect of eAdo on eATP mediated activation in vitro of Nlrp3 inflammasome in presence or absence A_2A_ and A_2B_ inhibitors evaluated by Caspase-Glo® 1 Inflammasome assay (Promega). BM MNC were isolated, and caspase 1 activation was measured. Experiments were repeated three times. ns – not significant, **p < 0.001. **Panel (B)** The effect of Ado, Ado plus A_2B_ inhibitor PSB1115 or A_2A_ inhibitor SCH442416 (10 µM of each) on the phosphorylation of the intracellular the nuclear factor (NF)-kappa B p65(RelA)^ser536^ intracellular pathway protein in treated murine bone marrow derived mononuclear cells was analyzed by western blotting. These cells (2 × 10^6^ cells per ml) were rendered quiescent for 3 h in RPMI medium containing 0.5% BSA at 37 °C, and afterwards the protein lysates were collected after 5 min of stimulation. Proteins retrieved from cells cultured in only assay medium were served as a control. **Panel (C)** Western blotting for expression of nuclear factor-like 2 (Nrf2) in protein lysates (40 µg of each sample) collected from pre-treated mononuclear cells. After incubation of cells with the target compounds at mentioned above concentrations, protein was immediately extracted and then quantified using the Pierce BCA Protein Assay Kit (Thermo Scientific, Rockford, IL, USA) and Multimode Analysis Software (Beckman Coulter Inc, Fullerton CA, USA). Expression of β-actin was also analyzed to ensure that all lanes were equivalently loaded. **Panel (D)** Effect of adenosine and its receptor antagonists on intracellular levels of cyclic AMP in pre-treated MNCs (4 × 10^4^ cells for each sample). cAMP concentrations were detected by cyclic AMP Assay kit (Cell signaling; #4339). *P < 0.05 is considered statistically significant between pre-treated and untreated cells. **Panel (E)** Expression of HO-1 in protein lysates collected from pre-treated mononuclear cells with adenosine with or without A_2B_ (PSB1115) or A_2A_ (SCH442416) inhibitor at the mentioned above concentrations for 1 h at 37^o^C. β-actin was also analyzed to ensure that the lanes were equivalently loaded. **Panel (F)** RT-qPCR analysis of murine HO-1 transcripts in mRNA samples that were purified from mouse MNCs, cultured with the tested compounds in a serum-free medium for 1 h at 37 °C. β2-microglobulin was used as an endogenous control. Samples containing only water instead of cDNA were used in each run as a negative control. Cells without any stimulation served as a control. *P < 0.05 is considered statistically significant between pre-treated and untreated cells

Based on our pervious data demonstrating an important role of Nlrp3 inflammasome in migration of HSPCs and negative effect of HO-1 on activation of this intracellular PRR [26, 29, 34], we investigated if this defect may be directly link to activation of A_2B_ receptor. To address this murine BMMNCs were exposed to eATP + eAdo in a presence of small molecule inhibitors of A_2A_ and A_2B_ receptors and we measured effect of eAdo on eATP promoted activation of of Nlrp3 inflammasome. We noticed that eAdo inhibits activation of Nlrp3 inflammasome, and that this inhibitory effect was mediated by A_2B_ receptor (Fig. 4A).

Next, based on our previous observation that eAdo upregulates HO-1 in BMMNC [6, 31] we become interested again which signaling pathways are involved in this effect. Based on data reported in smooth muscle cells we tested if upregulation of HO-1 in HSPCs is mediated by activation of cAMP. We noticed that that eAdo stimulates in murine BMMNC phosphorylation of NF-kBp65 (Fig. 4B), expression of Nrf2 transcription factor (Fig. 4C) and cAMP (Fig. 4D) that all combined are involved in upregulation of HO-1 (Fig. 4E and F). What is important this occurred in A_2B_-receptor dependent manner. Therefore, this data explains at molecular level eAdo mediated inhibition of trafficking of hematopoietic cells due to upregulation of HO-1 via A_2B_ receptor.

### CD39 and CD73 Ectonucleotidases are Involved in eAdo Mediated Regulation of Homing and Engraftment of HSPCs

As mentioned eAdo in extracellular space is generated from eATP by two major ectonucleotidases CD39 and CD73 that are expressed on surface of many cells including HSPCs and cells present in BM microenvironment [7, 35]. Our data presented in Fig. 3 indicates that eAdo could inhibit migration of HSPCs directly at level of HSPCs (Fig. 3B) and at level of hematopoietic microenvironment (Fig. 3C) as both ectonucleotidases are abundantly expressed on the cells.

**Fig. 5:**
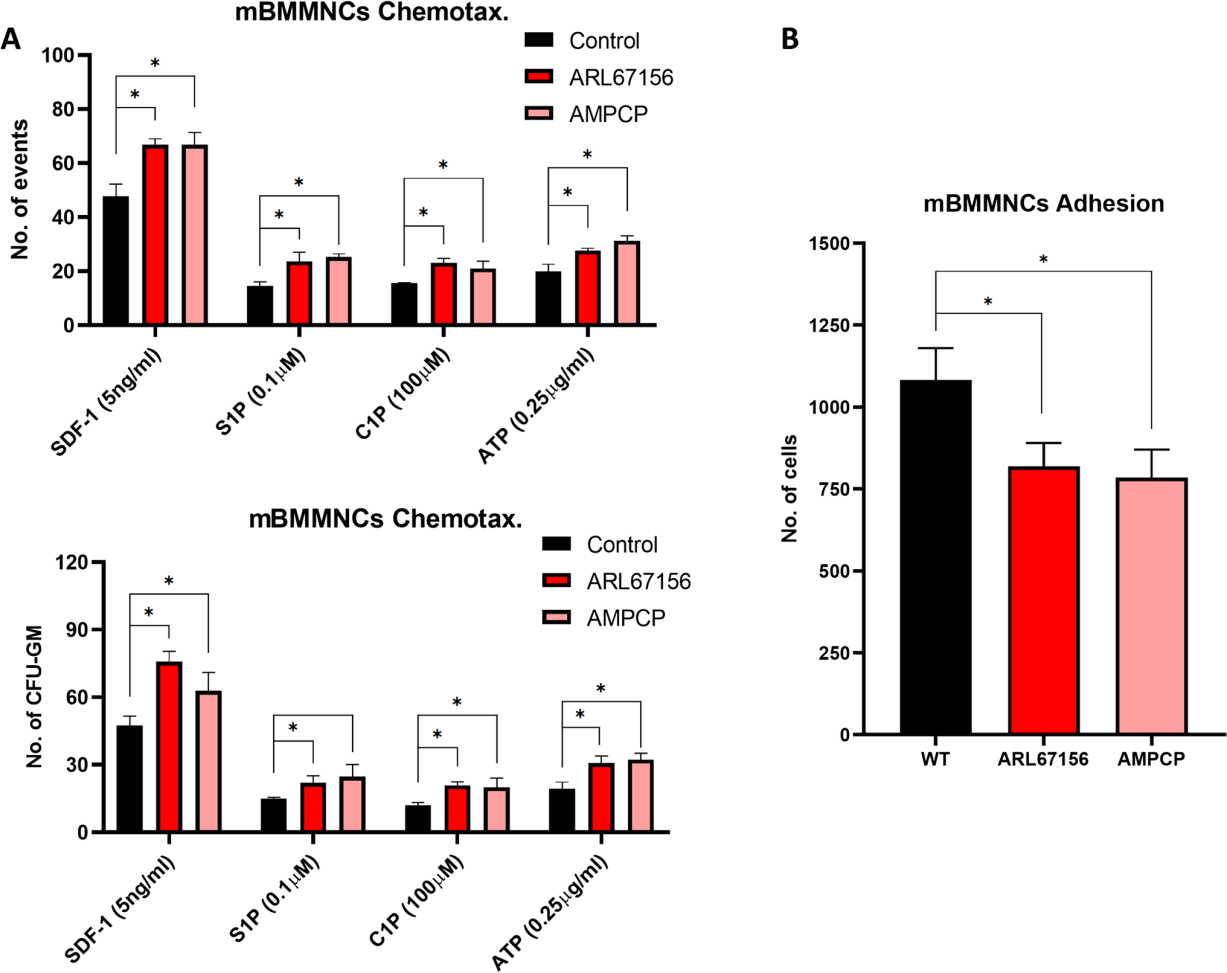
Inhibition of CD39 and C73 on HSPCs promotes their migration and adhesion. **Panel (A)** The chemotactic responsiveness to SDF-1, S1P, C1P, and ATP gradients of BMMNCs alone or treated with CD39 or CD73 inhibitors (respectively ARL67156 and AMPCP) by FACS and clonogenic CFU-GM progenitors. Results are combined from three independent experiments. *p > 0.05. **Panel (B)** The effect of CD39 and CD73 inhibitors on adhesion of murine BMMNCs to fibronectin- coated plates. The results are shown as the number of adherent cells. Data from four separate experiments are pooled together. *p < 0.01

In our previous work we have shown that inhibition of CD39 and CD73 by small molecular inhibitors of ectonucleotidases enhanced pharmacological mobilization of HSPCs [7]. To address a potential role of CD39 and CD73 inhibition on homing and engraftment of BMMNCs we exposed as first murine BMMNC to specific CD39 and CD73 inhibitors, ARL67156 and AMPCP respectively and tested in TransWell assay the migratory potential of these cells to major BM homing factors for HSPCs such as stromal-derived factor-1 (SDF-1), sphingosine-1 phosphate (S1P), ceramide-1 phosphate (C1P) and eATP (Fig. 5A) as well as tested their adhesive properties (Fig. 5B). We noticed that inhibition of both ectonucleotidases improves migration of murine BMMNCs and CFU-GM to these BM homing factors what indicates an involvement of autocrine secreted eATP-eAdo loop in migration and adhesion of HSPCs. While migration was improved, adhesion of BMMNCs was inhibited. This confirms that CD39 and CD73 inhibition promotes overall migratory potential of BMMNCs.

**Fig. 6:**
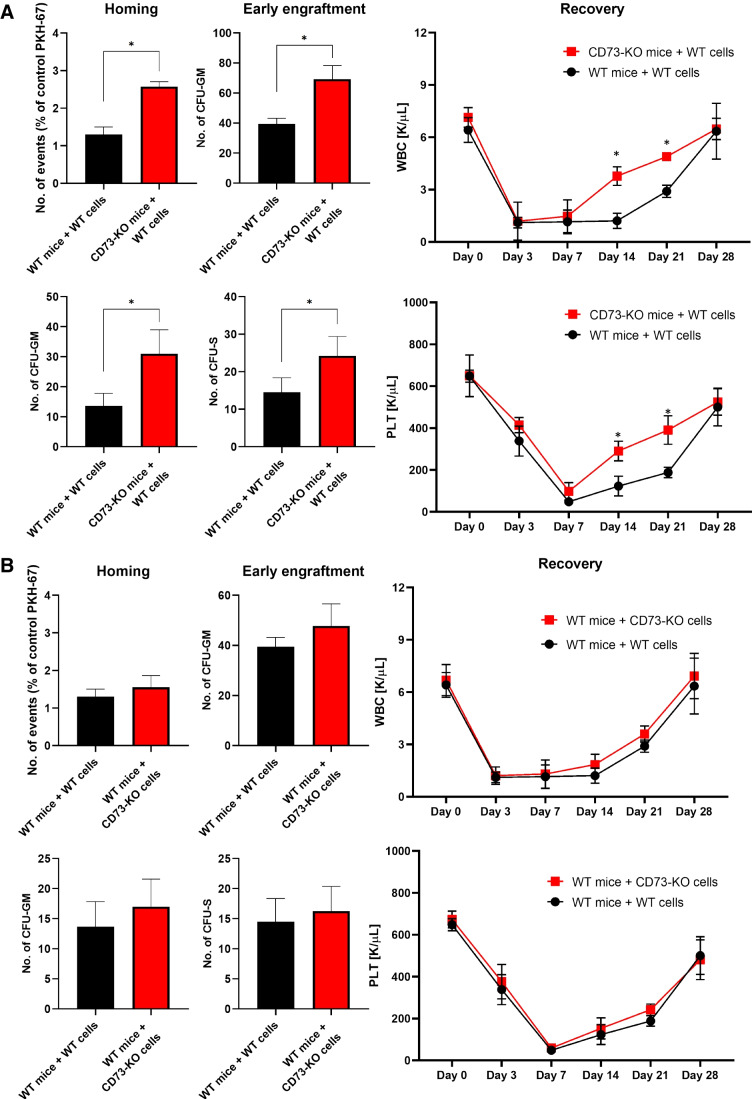
Lack of CD73 on HSPCs or in BM microenvironment improves homing and engraftment of HSPCs. **Panel A.** 1.5 × 10^5^ BMMNCs derived from WT mice were transplanted into lethally irradiated CD73-KO or WT animals. The lack of CD73 in BM microenvironment promoted both homing (**A** left panel), engraftment (**A** middle panel), and recovery of peripheral blood counts ((**A** right panel). Similar experiments we have performed using as BMMNCs recipients and BMMNCs isolated from CD73-KO or WT mice (**B**). Again we noticed slight but not statistically significant improvement in homing and early engraftment of transplanted CD73-KO BMMNCs. No colonies were formed in lethally irradiated, untransplanted mice (irradiation control). White blood cells (WBC) and platelets (PLT) were counted at intervals (at 0, 3, 7, 14, 21, and 28 days after transplantation). *p < 0.05

To provide more direct evidence we also performed transplantation using BMMNC-derived from WT mice and transplanted them into CD73-KO and WT animals (Fig. 6A) and noticed that lack of CD73 expression in recipient BM microenvironment promoted both homing (Fig. 6A left panel), early engraftment (Fig. 6A middle panel) and accelerated recovery of peripheral blood counts (Fig. 6A right panel). Similar experiments we performed using as BMMNCs recipients WT animals and BMMNCs isolated from CD73-KO and WT mice (Fig. 6B). Again we noticed slight improvement of homing and early engraftment of transplanted CD73-KO BMMNCs what indicates a role of HSPCs expressed CD73 in hematopoietic reconstitution after transplantation.

### Proteomic Analysis of eAdo Stimulated Murine SKL Cells in Presence or Absence of A_2B_ and A_2A_ Inhibitors

**Fig. 7:**
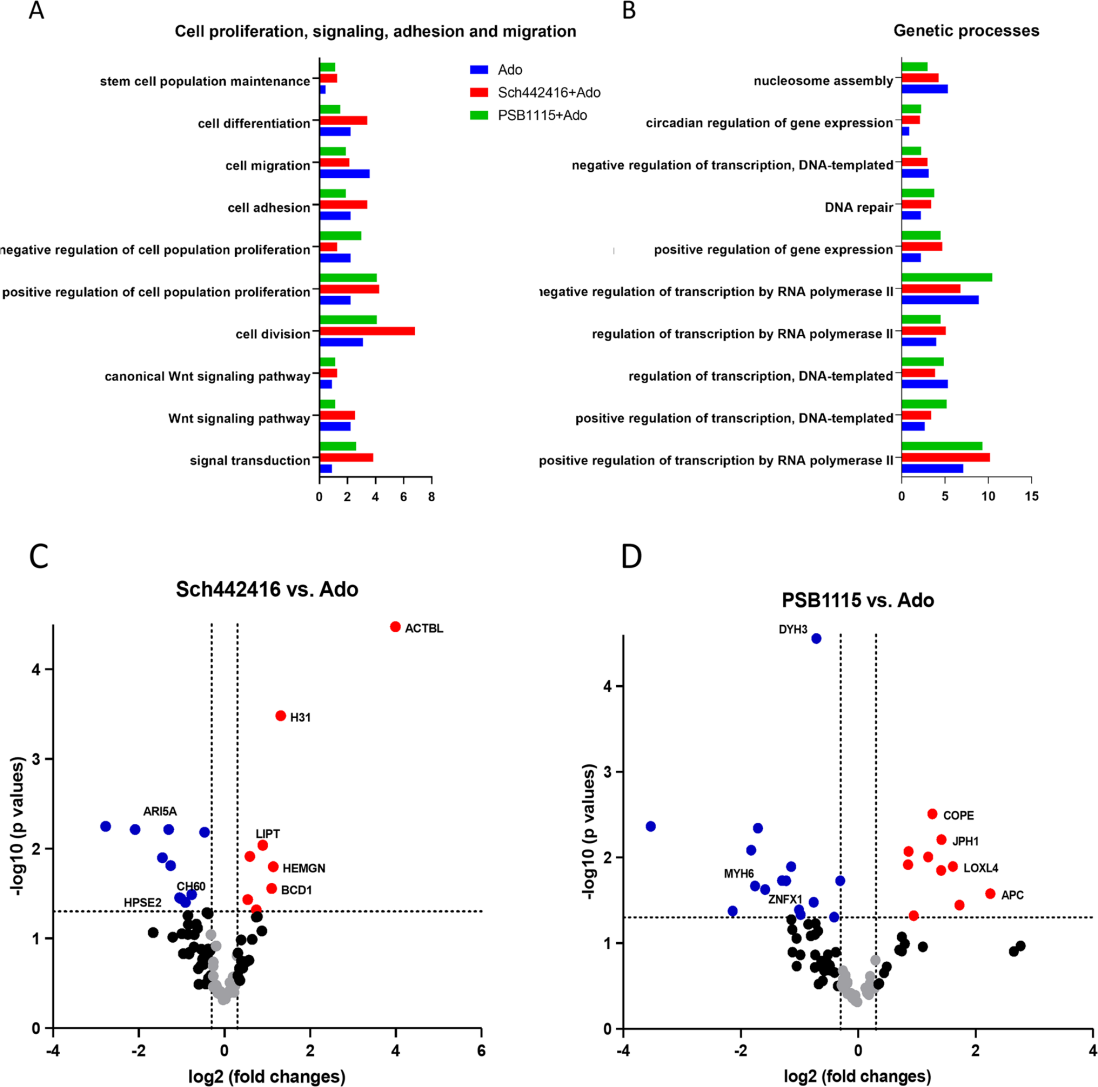
The proteomic profile of murine SKL (Sca-1^+^/c-Kit^+^/Lin^−^) cells after A_2A_ and A_2B_ adenosine receptors inhibition.** Panel (A)** Functional classification of gene orthology (GO) of proteomic data in Ado, SCH442416 + Ado and PSB1115 + Ado datasets. Comparative analysis of annotated proteins corresponding to GO biological processes regulating cell proliferation, cell signaling, adhesion, and migration in all three datasets. **Panel (B)** Functional classification of gene orthology (GO) of proteomic data in Ado, SCH442416 + Ado and PSB1115 + Ado datasets. Comparative analysis of annotated proteins corresponding to GO biological processes regulating transcription and gene expression in all three datasets. **Panel (C)** Volcano plots displaying the distribution of all common proteins in dataset SCH442416 + Ado vs. Ado (n = 110) with relative protein abundance (log2 fold change) plotted against its significance level (negative log10 P-value), showing significantly (P < 0.05) increased (red dots) and decreased (blue dots) proteins in SKL cells after A_2A_ or A_2B_ receptors inhibition co-stimulated with Ado. **Panel (D)** Volcano plots displaying the distribution of all common proteins in dataset PSB1115 + Ado vs. Ado (n = 95) with relative protein abundance (log2 fold change) plotted against its significance level (negative log10 P-value), showing significantly (P < 0.05) increased (red dots) and decreased (blue dots) proteins in SKL cells after A_2A_ or A_2B_ receptors inhibition and cell co-stimulated with Ado

Analyzing GO biological processes we noticed increase in number of proteins involved in cell proliferation, cell signaling and transcription after A_2A_ and A_2B_ inhibition (Fig. 7A and B). We noticed also that inhibition of A_2B_ receptor led to statistically significant up-regulation of adenomatous polyposis coli protein APC (4.8 fold, p = 0.026), a key player of Wnt signaling pathways required for cell polarization and directional migration [36], as well as in up-regulation of transcriptional factor SOX6 [37] (1.8 times) and 1.9 fold up-regulation of a nuclear receptor binding factor-2 (NRBF-2) [38]. At the same time we observed a 1.9 times down-regulation in expression of nuclear receptor coactivator 3 (Ncoa-3) [39] (Fig. 7D).

Interestingly, despite a fact that the number of all annotated proteins involved in cell migration and adhesion was higher in Ado dataset, we find that A_2B_ inhibition led to up-regulation of proteins that positively promote migration. Accordingly, we noticed increase in lysyl oxidase like 4 (Loxl4) [40–42] up to 3.1, junctophilin-1 (JPH1) [43] up to 2.7 times, girdin [44] up to 1.6 times as compared to Ado treatment alone. In addition, after Ado treatment we detected increase in SKL cells of adhesion proteins including filamin A [45], junction plakoglobin [46], talin-1 [47], desmoplakin [48] and ADAM15 [49]. At the same time, after A_2A_ and A_2B_ inhibition we detected proteins annotated in GO that are involved in cell adhesion and function as regulators of actin cytoskeleton dynamics and extracellular matrix rearrangement. We observed also significant down-regulation of expression of minus-ended motors: dynein axonemal heavy chain 3 [50] (1.6 times) and unconventional myosin 6 [51] (3.4 times) in cells exposed to A_2B_ inhibitor as compared to Ado treatment only. Overall, this proteomics signature suggests that A_2B_ inhibition promotes migration and proliferation of murine HSPCs.

## Discussion

The seminal observation of this paper is that eAdo inhibits in A_2B_-HO-1-Nlrp3 inflammasome-dependent manner mobilization, migration, homing and engraftment of HSPCs. This eAdo negative effect on Nlrp3 inflammasome requires A_2B_ mediated increase of intracellular anti-inflammatory level of HO-1. Therefore, we provide further evidence that pharmacological mobilization or conditioning for transplantation by lethal irradiation induces sterile inflammation in BM microenvironment which by integrating purinergic signaling and innate immunity responses promotes in eATP-dependent manner trafficking of HSPCs, and that this effect is negatively regulated by eATP metabolite eAdo that activates A_2B_ receptor [6, 7].

Our previous experiments revealed a pivotal role of eATP mediated activation of Nlrp3 inflammasome in migration of HSPCs [6, 22, 24]. One of the mechanisms responsible for this phenomenon was Nlrp3 inflammasome promoted incorporation of CXCR4 homing receptor for SDF-1 into membrane lipid rafts (MLRs) [23]. The presence of CXCR4 in MLRS on migrating cells ensures as reported their optimal responsiveness to SDF-1 [23, 52], and eAdo as we have shown inhibits formation of MLRs [23]. Nevertheless, type of eAdo receptor responsible for this phenomenon has not been identified in our previous work.

Therefore, we asked which of cell surface P1 purinergic receptors is responsible for eAdo-mediated defective cell mobilization, migration, homing and engraftment of HSPCs. It was an important question to ask since small molecular safe for use in vivo inhibitors of these receptors are commercially available. We report herein that murine as well as human HSPCs express highly two P1 receptors – A_2A_ and A_2B_ and that A_2B_ turned out to be critical for negative effects of eAdo on HSPCs trafficking. Interestingly, A_2B_ receptor has been reported to be involved in initiation of hematopoiesis in zebra fish [15] and to play and evolutionary conserved role in the first steps of hematopoietic stem progenitor cells (HSPCs) formation in vertebrates [15, 19, 53]. Therefore, our data, add new information that A_2B_ receptor inhibits migration and increases adhesion of adult HSPCs.

We also found that eAdo primarily is involved in trafficking of HSPCs and does not affect proliferation of clonogeneic progenitors (data not shown). It is somehow in contrast to previous reports indicating that in adult mice two other eAdo P1 receptors – namely A_1_ and A_3_ play a homeostatic opposite role in suppressing or stimulating hematopoiesis, respectively [2, 16, 18]. It has been also postulated that selective activation of A_3_ receptors promoted hematopoiesis by acting on more differentiated hematopoietic progenitor cells. These differences between our and other data in vitro could be explained by differences in murine strains employed.

Nevertheless, our data clearly shows that eAdo exogenously administrated into lethally irradiated mice has negative effect on speed of hematopoietic reconstitution of mice transplanted with WT BMMNCs. Moreover, blockage of A_2B_ receptor on transplanted cells improved their mobilization, homing, engraftment and accelerates hematopoietic reconstitution. Therefore, in our hands eAdo is a negative regulator of trafficking of HSPCs. In support of this as reported eAdo inhibits also migration of lymphocytes [54]. However, the final effect of P1 receptors in cell migration and proliferation may depend on cell type employed in experimental settings. For example while eAdo inhibited motility of T cells and A_2B_ specific agonist BAY 60-6583 decreased migration of polymorphonuclear leucocytes [54, 55], eAdo enhanced migration of rhabdomyosarcoma cell lines [56] and stimulated growth of endothelial cells [57].

Overall, our data supports a concept that there are two sources of eAdo that may affect trafficking of HSPCs. The first source is eAdo processed in CD39 and CD73-dependent manner from eATP autocrine secreted from activated cells. The other source is an “eAdo cloud” present in BM microenvironment as result of pharmacological mobilization or myeloablative conditioning for transplant processed similarly in CD39 and CD73-dependent manner from eATP released from stressed cells. It is why exposure of BMMNC before mobilization or transplantation to CD39 and CD73 small molecular inhibitors, as well as preconditioning of mobilized mice or lethally irradiated for transplant with these inhibitors decreases available eAdo and has a positive effect on mobilization, homing, engraftment and speed of hematopoietic recovery after transplantation. Based on this small molecular inhibitors of CD39 and CD73 could find practical application in improving transplant protocols [6, 7].

In this current work we demonstrated that eAdo by activating A_2B_ receptor leads to increase in intracellular level of cAMP, Nrf2 that upregulate intracellular level of HO-1 to inhibit Nlrp3 inflammasome. Nlrp3 inflammasome is required for optimal migration of HSPCs and its intracellular level is regulated by different activators and inhibitors. The most important activators are danger associated pattern molecules (DAMPs) such as eATP [6, 22], reactive oxygen species (ROS) [58] or oxidized mitochondrial DNA [59]. On the other hand, Nlrp3 inflammasome inhibitors include some miRNA [60] and lncRNA species [61] as well as intracellular HO-1 [34]. While NRF2 is well established transcription factor that regulates the expression of antioxidant proteins to protect against oxidative damage triggered by injury and inflammation and induces HO-1 expression [11, 62], cAMP has been reported to increase expression of HO-1 in vascular cells [10] and hepatocytes [63]. In our current work we noticed that HO-1 is upregulated in BMMNCs in cAMP and Nrf2 dependent manner and inhibits Nlrp3 inflammasome in HSPCs, similarly as observed in non-hematopoietic cells. This explains at molecular level eAdo-A_2B_ mediated inhibition on trafficking of these cells. Moreover, since eAdo upregulates via A_2B_ receptor HO-1 in HSPCs and BM microenvironment cells, inhibition of HO-1 may additionally improve mobilization, homing and engraftment of HSPCs [26, 31].

To support our in vitro and in vivo observations we noticed that the proteomic profile of murine HSPCs after A_2B_ inhibition is characterized by up-regulation of proteins promoting cell proliferation, transcription and migration. The up-regulated lysyl oxidase like 4 (Loxl4) that belongs to LOX family is known to play a critical role in ECM formation [40, 42], stabilization and repair by contributing to the biogenesis of collagen and elastin [40, 41]. Moreover, we observed that inhibition of A_2B_ receptor led to up-regulation of adenomatous polyposis coli protein (APC), a key player of Wnt signaling pathways required for cell polarization and directional migration [36]. APC plays also a critical role in HSPCs self-renewal by inhibition of β-catenin–promoting proliferation and apoptosis [64]. We also noticed a significant up-regulation of transcriptional factor SOX6, that plays a key role in several developmental processes and up-regulation of a transcriptional activator and autophagy suppressor: nuclear receptor binding factor-2 (NRBF-2) [37, 38]. Such proteomics signature suggests that A_2B_ inhibition promotes migration and proliferation of HSPCs.

Base on this our data is highly relevant for potential optimization of HSPCs transplant strategies. The outcome of hematopoietic transplantation depends widely on number of cells available for transplant as well as speed of engraftment of these cells in recipient. It is known that not always hematopoietic graft provides an optimal number of cells [65, 66]. For example it could be result of (i) poor harvest of HSPCs after pharmacological mobilization of the donor, (ii) suboptimal collection of these cells from the donor BM, and (iii) finally umbilical cord blood intendent for transplantation may contain low number of cells for an adult graft recipient. Therefore, improving mobilization of HSPCs similarly as improving homing/seeding efficiency of transplanted cells is an important task for satisfactory transplant outcomes. Furthermore, since Nlrp3 inflammasome regulates aging-associated chronic inflammation of HSPCs it would be interesting to see effect of A2b signaling in this phenomenon [67].

In conclusion we postulate that modification of purinergic signaling in HSPCs and BM microenvironment by inhibiting negative effects of eAdo-A_2B_ axis could become an important strategy for facilitating mobilization, homing and engraftment of transplanted HSPCs. Thus, our data obtained in mice if verified in humans could help by employing small inhibitors of A_2B_ to improve HSPCs transplant outcomes.

## Supplementary Information

Below is the link to the electronic supplementary material.

Supplementary Fig. 1 Blockage of the A_2A_ receptor does not affect homing and engraftment of HSPCs. (**Panel A**) Data shows that the A_2A_ receptor antagonist, SCH442416 does not improve early homing. Mice were irradiated (1000 cGY) 24 h before transplanting BMMNCs from WT mice (5 × 10^6^/100 µl/mice, labeled with PKH-67) and treated or untreated with A_2a_ inhibitor (SCH442416; 10 µM). Next day, homing was evaluated by; PKH-67 FACS analysis (top) and a number of clonogenic CFU-GM (bottom). (**Panel B**) Mice were injected with A_2a_ inhibitor (SCH442416; 3 mg/kg daily for 7 days) prior to the irradiation procedure and transplanted (5 × 10^6^/100 µl/mice) with BMMNCs isolated from WT mice. Homing was evaluated by; PKH-67 FACS analysis (top) and a number of clonogenic CFU-GM culture assays (bottom) as described in Material and Methods. (PNG 56 kb)

High Resolution Image (TIFF 2662 kb)

Supplementary Fig. 2 A_2B_ receptor more specific agonist BAY60.6083 inhibits homing and engraftment of HSPCs. Mice were mobilized with AMD3100 (5 mg/kg, once) with eAdo (3 mg/kg, 4 doses) or BAY 60-6083 (3 mg/kg, 4 doses). 1 h after injections PB was isolated and a number of WBC (left panel), circulating SKL cells (middle panel) and clonogenic CFU-GM (right panel) were evaluated. *p < 0.05. (PNG 54 kb)

High Resolution Image (TIFF 2662 kb)
