# Supplementary figures and images for: Extracellular Adenosine (eAdo) - A2B Receptor Axis Inhibits in Nlrp3 Inflammasome-dependent Manner Trafficking of Hematopoietic Stem/progenitor Cells
Source: Stem Cell Rev Rep. 2022 Jul 23;18(8):2893–911. doi: 10.1007/s12015-022-10417-w (PMC9622533; doi:10.1007/s12015-022-10417-w)

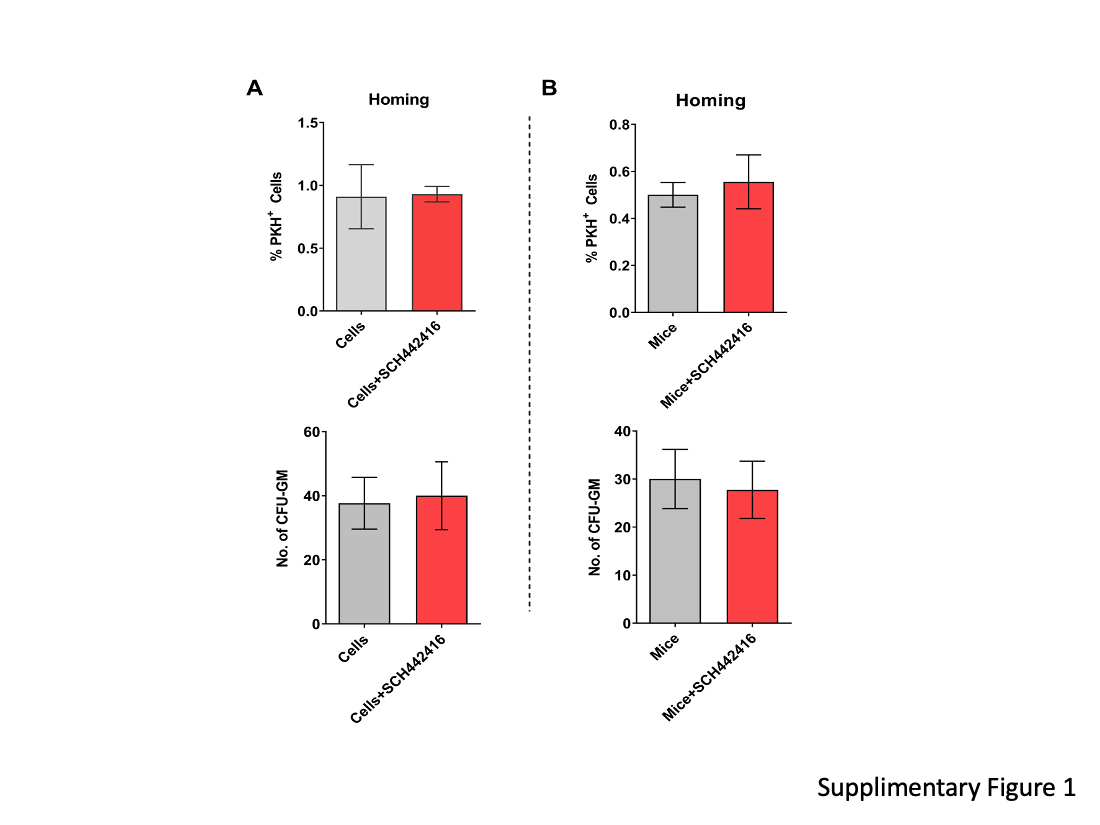

Supplement: Supplementary file 1 — Blockage of the A2A receptor does not affect homing and engraftment of HSPCs. (Panel A) Data shows that the A2A receptor antagonist, SCH442416 does not improve early homing. Mice were irradiated (1000 cGY) 24 h before transplanting BMMNCs from WT mice (5 × 106/100 µl/mice, labeled with PKH-67) and treated or untreated with A2a inhibitor (SCH442416; 10 µM). Next day, homing was evaluated by; PKH-67 FACS analysis (top) and a number of clonogenic CFU-GM (bottom). (Panel B) Mice were injected with A2a inhibitor (SCH442416; 3 mg/kg daily for 7 days) prior to the irradiation procedure and transplanted (5 × 106/100 µl/mice) with BMMNCs isolated from WT mice. Homing was evaluated by; PKH-67 FACS analysis (top) and a number of clonogenic CFU-GM culture assays (bottom) as described in Material and Methods. (PNG 56 kb) [file 12015_2022_10417_Fig8_ESM.png]

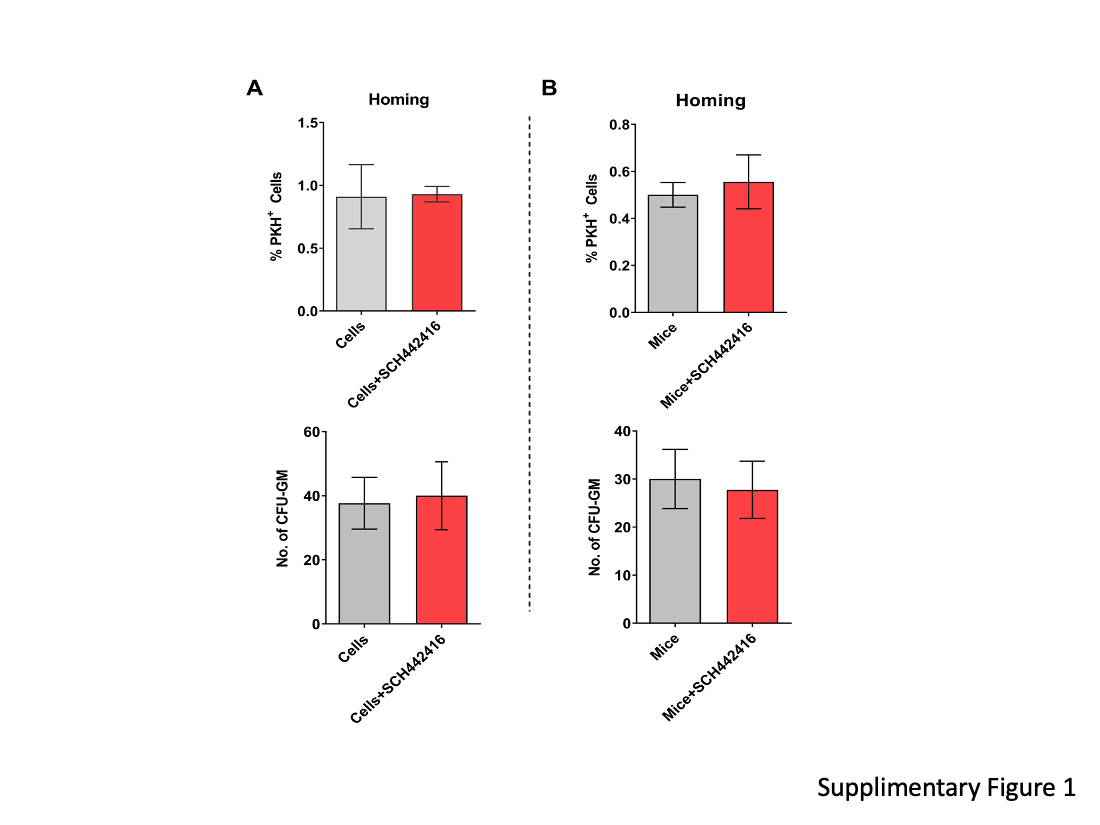

Supplement: Supplementary file 2 — High Resolution Image (TIFF 2662 kb) [file 12015_2022_10417_MOESM1_ESM.tiff]

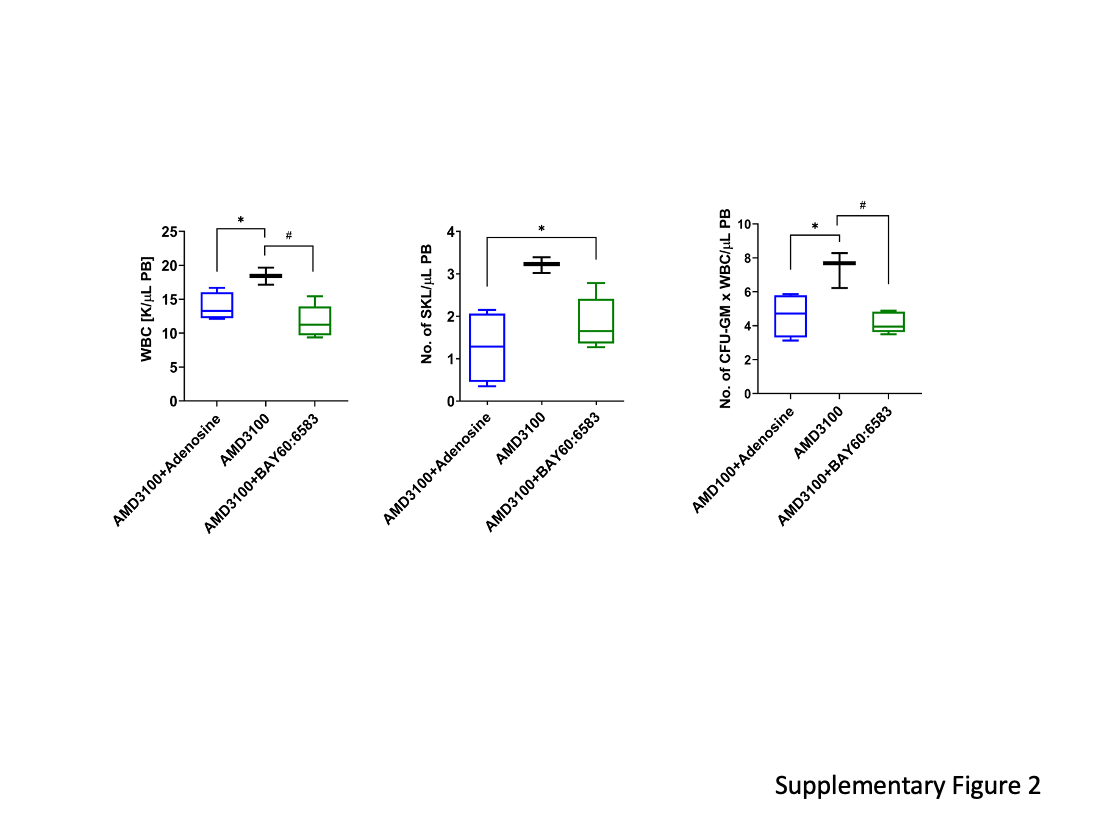

Supplement: Supplementary file 3 — A2B receptor more specific agonist BAY60.6083 inhibits homing and engraftment of HSPCs. Mice were mobilized with AMD3100 (5 mg/kg, once) with eAdo (3 mg/kg, 4 doses) or BAY 60-6083 (3 mg/kg, 4 doses). 1 h after injections PB was isolated and a number of WBC (left panel), circulating SKL cells (middle panel) and clonogenic CFU-GM (right panel) were evaluated. *p < 0.05. (PNG 54 kb) [file 12015_2022_10417_Fig9_ESM.png]

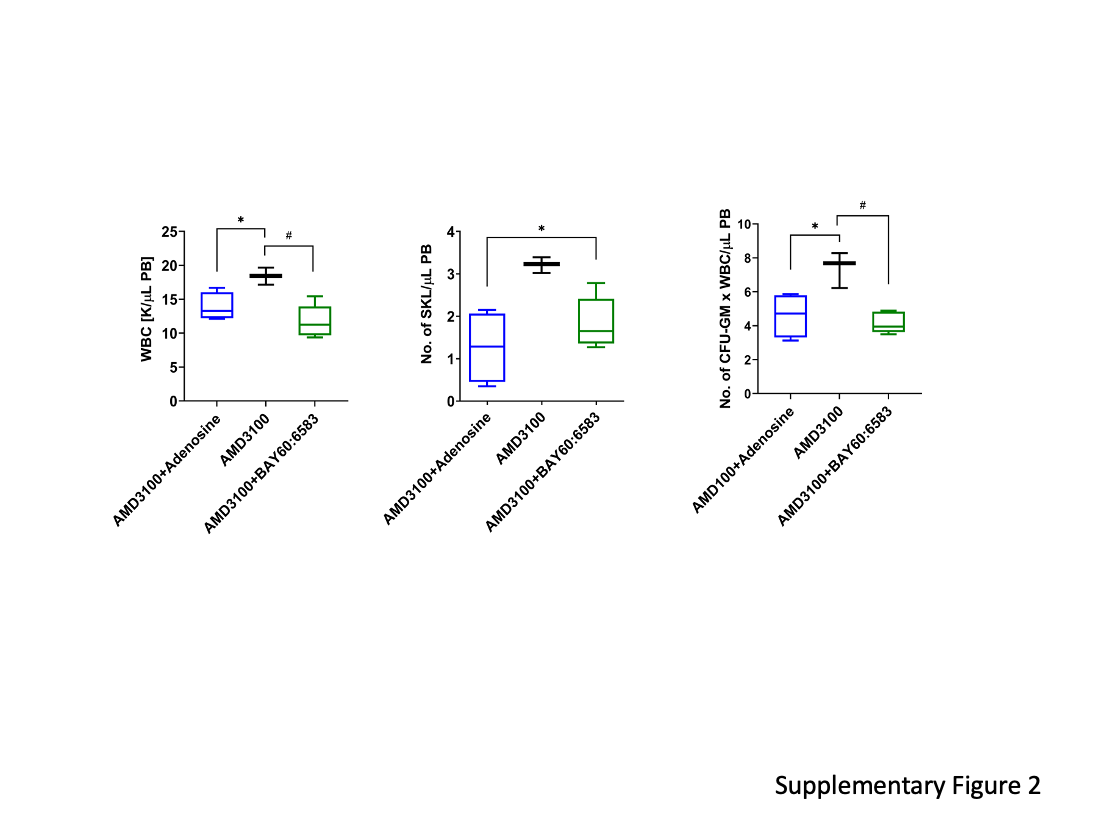

Supplement: Supplementary file 4 — High Resolution Image (TIFF 2662 kb) [file 12015_2022_10417_MOESM2_ESM.tiff]
